# 1-(2,4-Dichloro­phen­yl)-3-[4-(dimethyl­amino)phen­yl]prop-2-enone

**DOI:** 10.1107/S1600536809015177

**Published:** 2009-05-14

**Authors:** Navin N. Bappalige, Brinda, Y. Narayana, V. Upadyaya

**Affiliations:** a Department of Physics, Mangalore University, Mangalagangothri, Mangalore 574 199, India; b Solid State and Structural Chemistry Unit, Indian Institute of Science, Bangalore 560 012, India; c Department of Physics, Manipal Institute of Technology, Manipal 576 104, India

## Abstract

In the title compound, C_17_H_15_Cl_2_NO, the dimethyl­amino­phenyl group is close to coplanar with the central propenone group [dihedral angle = 13.1 (1)° between the mean planes], while the dichloro­phenyl group is twisted from the plane [dihedral angle = 64.0 (1)°]. In the crystal, C—H⋯O and weak C—H⋯π inter­actions are formed between mol­ecules.

## Related literature

For related structures, see: Murafuji *et al.* (1999 ▶); Liu *et al.* (2002 ▶); Patil *et al.* (2007*a* ▶,*b* ▶); Rosli *et al.* (2007 ▶).
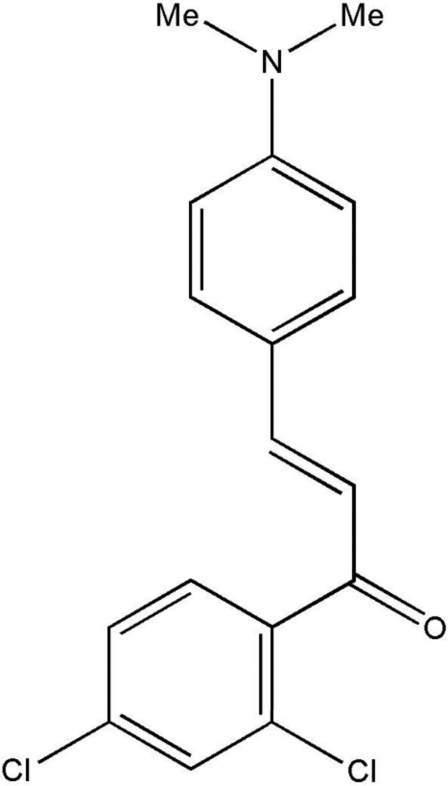


## Experimental

### 

#### Crystal data

C_17_H_15_Cl_2_NO
*M* *_r_* = 320.20
Monoclinic, 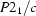

*a* = 8.5741 (19) Å
*b* = 12.706 (3) Å
*c* = 14.671 (3) Å
β = 102.645 (4)°
*V* = 1559.5 (6) Å^3^
*Z* = 4
Mo *K*α radiation
μ = 0.41 mm^−1^
*T* = 290 K
0.25 × 0.15 × 0.07 mm

#### Data collection

Bruker SMART APEX CCD area-detector diffractometer
Absorption correction: multi-scan (*SADABS*; Sheldrick, 1996 ▶) *T*_min_ = 0.923, *T*_max_ = 0.972
11540 measured reflections
2908 independent reflections
2039 reflections with *I* > 2σ(*I*)
*R*_int_ = 0.031

#### Refinement

*R*[*F* ^2^ > 2σ(*F* ^2^)] = 0.042
*wR*(*F* ^2^) = 0.106
*S* = 1.03
2908 reflections
192 parameters
H-atom parameters constrained
Δρ_max_ = 0.20 e Å^−3^
Δρ_min_ = −0.17 e Å^−3^

### 

Data collection: *SMART* (Bruker, 2004 ▶); cell refinement: *SAINT* (Bruker, 2004 ▶); data reduction: *SAINT*; program(s) used to solve structure: *SHELXS97* (Sheldrick, 2008 ▶); program(s) used to refine structure: *SHELXL97* (Sheldrick, 2008 ▶); molecular graphics: *ORTEP-3 for Windows* (Farrugia, 1997 ▶) and *CAMERON* (Watkin *et al.*, 1993 ▶); software used to prepare material for publication: *PLATON* (Spek, 2009 ▶).

## Supplementary Material

Crystal structure: contains datablocks global, I. DOI: 10.1107/S1600536809015177/bi2360sup1.cif

Structure factors: contains datablocks I. DOI: 10.1107/S1600536809015177/bi2360Isup2.hkl

Additional supplementary materials:  crystallographic information; 3D view; checkCIF report

## Figures and Tables

**Table 1:** Hydrogen-bond geometry (Å, °)

*D*—H⋯*A*	*D*—H	H⋯*A*	*D*⋯*A*	*D*—H⋯*A*
C12—H12⋯O1^i^	0.93	2.55	3.252 (3)	132
C4—H4⋯*Cg*1^ii^	0.93	2.95	3.784 (3)	150

Symmetry codes: (i) 
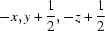
; (ii) 
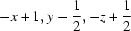
. *Cg*1 is the centroid of the C10–C15 ring.

**Fig. 1.**
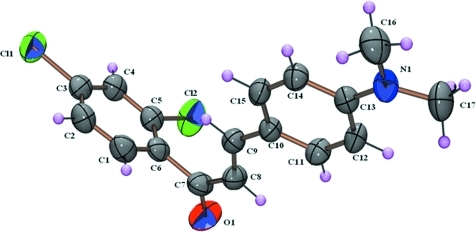
The molecular structure with displacement ellipsoids drawn at the 50% probability level for non-H atoms. H atoms are shown as small spheres of arbitrary radius.

**Fig. 2.**
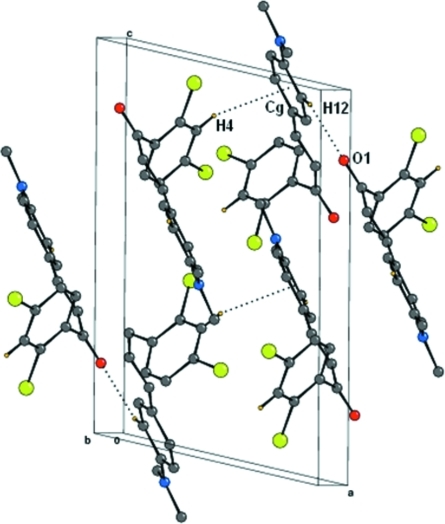
Packing diagram. The dotted lines indicate intermolecular C—H···O and C—H···π interactions.

## supplementary crystallographic information

### Experimental

A solution of potassium hydroxide (6.25 g, 0.11 mol) in ethanol (25 ml) was added slowly to a mixture of dichloroacetophenone (18.8 g, 0.01 mol) and *N*-dimethyl benzaldehyde (14.9 g, 0.01 mol) in a conical flask. After stirring for two hours, the precipitate was filtered and recrystallized from ethanol to give pale orange crystals.

### Refinement

H atoms were positioned geometrically with C—H bond lengths of 0.93–0.96 Å and refined using a riding model with *U*_iso_(H) = 1.2 or 1.5*U*_eq_(C).

### Crystal data

**Table:**

C_17_H_15_Cl_2_NO	*F*(000) = 664
*M**_r_* = 320.20	*D*_x_ = 1.364 Mg m^−^^3^
Monoclinic, *P*2_1_/*c*	Mo *K*α radiation, λ = 0.71073 Å
Hall symbol: -P 2ybc	Cell parameters from 3980 reflections
*a* = 8.5741 (19) Å	θ = 2.0–26.0°
*b* = 12.706 (3) Å	µ = 0.41 mm^−^^1^
*c* = 14.671 (3) Å	*T* = 290 K
β = 102.645 (4)°	Block, orange
*V* = 1559.5 (6) Å^3^	0.25 × 0.15 × 0.07 mm
*Z* = 4	

### Data collection

**Table:**

Bruker SMART APEX CCD area-detector diffractometer	2908 independent reflections
Radiation source: fine-focus sealed tube	2039 reflections with *I* > 2σ(*I*)
graphite	*R*_int_ = 0.031
φ and ω scans	θ_max_ = 25.5°, θ_min_ = 2.1°
Absorption correction: multi-scan (*SADABS*; Sheldrick, 1996)	*h* = −10→10
*T*_min_ = 0.923, *T*_max_ = 0.972	*k* = −15→15
11540 measured reflections	*l* = −16→17

### Refinement

**Table:**

Refinement on *F*^2^	Primary atom site location: structure-invariant direct methods
Least-squares matrix: full	Secondary atom site location: difference Fourier map
*R*[*F*^2^ > 2σ(*F*^2^)] = 0.042	Hydrogen site location: inferred from neighbouring sites
*wR*(*F*^2^) = 0.106	H-atom parameters constrained
*S* = 1.03	*w* = 1/[σ^2^(*F*_o_^2^) + (0.0505*P*)^2^ + 0.1972*P*] where *P* = (*F*_o_^2^ + 2*F*_c_^2^)/3
2908 reflections	(Δ/σ)_max_ < 0.001
192 parameters	Δρ_max_ = 0.20 e Å^−^^3^
0 restraints	Δρ_min_ = −0.17 e Å^−^^3^

### Special details

**Table:**

Geometry. All e.s.d.'s (except the e.s.d. in the dihedral angle between two l.s. planes) are estimated using the full covariance matrix. The cell e.s.d.'s are taken into account individually in the estimation of e.s.d.'s in distances, angles and torsion angles; correlations between e.s.d.'s in cell parameters are only used when they are defined by crystal symmetry. An approximate (isotropic) treatment of cell e.s.d.'s is used for estimating e.s.d.'s involving l.s. planes.
Refinement. Refinement of *F*^2^ against ALL reflections. The weighted *R*-factor *wR* and goodness of fit *S* are based on *F*^2^, conventional *R*-factors *R* are based on *F*, with *F* set to zero for negative *F*^2^. The threshold expression of *F*^2^ > σ(*F*^2^) is used only for calculating *R*-factors(gt) *etc*. and is not relevant to the choice of reflections for refinement. *R*-factors based on *F*^2^ are statistically about twice as large as those based on *F*, and *R*- factors based on ALL data will be even larger.

### Fractional atomic coordinates and isotropic or equivalent isotropic displacement parameters (Å^2^)

**Table:**

	*x*	*y*	*z*	*U*_iso_*/*U*_eq_	
Cl1	0.42606 (8)	−0.03118 (5)	0.21634 (5)	0.0704 (2)	
Cl2	0.33039 (10)	0.29372 (6)	0.42942 (5)	0.0851 (3)	
C13	0.2618 (2)	0.71607 (15)	−0.03057 (14)	0.0421 (5)	
C10	0.1972 (2)	0.54646 (15)	0.08139 (14)	0.0414 (5)	
C9	0.1721 (2)	0.45849 (17)	0.13874 (15)	0.0461 (5)	
H9	0.2244	0.3964	0.1299	0.055*	
C15	0.2754 (2)	0.53119 (16)	0.00809 (15)	0.0459 (5)	
H15	0.3078	0.4636	−0.0037	0.055*	
C8	0.0829 (3)	0.45512 (17)	0.20297 (16)	0.0508 (6)	
H8	0.0247	0.5151	0.2101	0.061*	
N1	0.2898 (2)	0.79802 (13)	−0.08543 (13)	0.0518 (5)	
C12	0.1844 (3)	0.73163 (16)	0.04341 (14)	0.0481 (5)	
H12	0.1548	0.7993	0.0568	0.058*	
C6	0.1595 (2)	0.26655 (16)	0.25224 (16)	0.0469 (5)	
C3	0.3204 (3)	0.08209 (17)	0.23089 (17)	0.0509 (6)	
C14	0.3057 (2)	0.61262 (16)	−0.04685 (14)	0.0456 (5)	
H14	0.3561	0.599	−0.0957	0.055*	
C4	0.3608 (3)	0.13662 (17)	0.31339 (16)	0.0519 (6)	
H4	0.4425	0.1129	0.3616	0.062*	
C11	0.1517 (2)	0.64926 (17)	0.09597 (15)	0.0476 (5)	
H11	0.0972	0.6622	0.1431	0.057*	
C7	0.0693 (3)	0.36611 (18)	0.26236 (17)	0.0547 (6)	
O1	−0.0115 (2)	0.37083 (15)	0.32101 (14)	0.0849 (6)	
C5	0.2780 (3)	0.22710 (17)	0.32357 (15)	0.0501 (5)	
C2	0.2015 (3)	0.11710 (19)	0.15886 (17)	0.0576 (6)	
H2	0.1737	0.079	0.1036	0.069*	
C17	0.2722 (3)	0.90604 (18)	−0.05879 (18)	0.0685 (7)	
H17A	0.1625	0.9193	−0.0574	0.103*	
H17B	0.304	0.9521	−0.1033	0.103*	
H17C	0.3383	0.9185	0.0021	0.103*	
C16	0.3630 (3)	0.77905 (19)	−0.16414 (17)	0.0665 (7)	
H16A	0.4725	0.7587	−0.1417	0.1*	
H16B	0.3584	0.8422	−0.2006	0.1*	
H16C	0.3064	0.7238	−0.2022	0.1*	
C1	0.1244 (3)	0.20911 (18)	0.16968 (17)	0.0577 (6)	
H1	0.046	0.2339	0.1202	0.069*	

### Atomic displacement parameters (Å^2^)

**Table:**

	*U*^11^	*U*^22^	*U*^33^	*U*^12^	*U*^13^	*U*^23^
Cl1	0.0823 (5)	0.0539 (4)	0.0800 (5)	0.0084 (3)	0.0288 (4)	0.0038 (3)
Cl2	0.1090 (6)	0.0798 (5)	0.0577 (4)	0.0164 (4)	−0.0008 (4)	−0.0118 (3)
C13	0.0447 (12)	0.0425 (12)	0.0377 (11)	−0.0018 (9)	0.0062 (9)	0.0013 (9)
C10	0.0437 (11)	0.0407 (12)	0.0406 (12)	0.0008 (9)	0.0110 (10)	0.0024 (9)
C9	0.0442 (12)	0.0439 (12)	0.0499 (13)	0.0026 (9)	0.0098 (10)	0.0023 (10)
C15	0.0531 (13)	0.0363 (11)	0.0508 (13)	0.0034 (10)	0.0166 (11)	−0.0031 (10)
C8	0.0476 (12)	0.0472 (13)	0.0604 (15)	0.0057 (10)	0.0178 (11)	0.0116 (11)
N1	0.0671 (12)	0.0415 (10)	0.0496 (11)	−0.0017 (9)	0.0187 (9)	0.0063 (8)
C12	0.0629 (14)	0.0365 (11)	0.0466 (13)	0.0078 (10)	0.0158 (11)	0.0002 (9)
C6	0.0451 (12)	0.0426 (12)	0.0546 (14)	−0.0054 (10)	0.0142 (11)	0.0104 (10)
C3	0.0543 (14)	0.0443 (13)	0.0585 (15)	−0.0036 (10)	0.0219 (12)	0.0083 (11)
C14	0.0524 (13)	0.0448 (12)	0.0430 (12)	0.0010 (10)	0.0176 (10)	−0.0010 (10)
C4	0.0545 (13)	0.0502 (13)	0.0496 (14)	0.0017 (11)	0.0081 (11)	0.0124 (11)
C11	0.0556 (13)	0.0496 (13)	0.0413 (12)	0.0050 (10)	0.0189 (10)	0.0009 (10)
C7	0.0451 (12)	0.0573 (14)	0.0645 (15)	−0.0020 (11)	0.0181 (12)	0.0118 (12)
O1	0.0906 (13)	0.0778 (13)	0.1068 (15)	0.0194 (10)	0.0665 (12)	0.0326 (11)
C5	0.0550 (13)	0.0484 (13)	0.0480 (13)	−0.0035 (11)	0.0135 (11)	0.0069 (10)
C2	0.0610 (15)	0.0550 (15)	0.0539 (14)	−0.0095 (12)	0.0061 (12)	−0.0027 (11)
C17	0.0926 (19)	0.0432 (14)	0.0702 (17)	−0.0039 (13)	0.0186 (15)	0.0076 (12)
C16	0.0852 (18)	0.0624 (16)	0.0571 (15)	−0.0086 (13)	0.0268 (14)	0.0111 (12)
C1	0.0508 (13)	0.0598 (15)	0.0562 (15)	−0.0032 (12)	−0.0019 (11)	0.0079 (12)

### Geometric parameters (Å, °)

**Table:**

Cl1—C3	1.738 (2)	C6—C1	1.389 (3)
Cl2—C5	1.739 (2)	C6—C7	1.507 (3)
C13—N1	1.369 (2)	C3—C4	1.372 (3)
C13—C14	1.402 (3)	C3—C2	1.372 (3)
C13—C12	1.405 (3)	C14—H14	0.930
C10—C11	1.393 (3)	C4—C5	1.376 (3)
C10—C15	1.400 (3)	C4—H4	0.9300
C10—C9	1.443 (3)	C11—H11	0.930
C9—C8	1.338 (3)	C7—O1	1.218 (3)
C9—H9	0.930	C2—C1	1.369 (3)
C15—C14	1.371 (3)	C2—H2	0.930
C15—H15	0.930	C17—H17A	0.960
C8—C7	1.448 (3)	C17—H17B	0.960
C8—H8	0.930	C17—H17C	0.960
N1—C17	1.444 (3)	C16—H16A	0.960
N1—C16	1.450 (3)	C16—H16B	0.960
C12—C11	1.365 (3)	C16—H16C	0.960
C12—H12	0.930	C1—H1	0.930
C6—C5	1.383 (3)		
			
N1—C13—C14	121.63 (18)	C3—C4—C5	118.8 (2)
N1—C13—C12	121.38 (18)	C3—C4—H4	120.6
C14—C13—C12	116.97 (18)	C5—C4—H4	120.6
C11—C10—C15	116.45 (18)	C12—C11—C10	122.21 (19)
C11—C10—C9	123.63 (19)	C12—C11—H11	118.9
C15—C10—C9	119.89 (18)	C10—C11—H11	118.9
C8—C9—C10	128.1 (2)	O1—C7—C8	121.3 (2)
C8—C9—H9	116.0	O1—C7—C6	119.6 (2)
C10—C9—H9	116.0	C8—C7—C6	119.03 (19)
C14—C15—C10	122.09 (19)	C4—C5—C6	122.1 (2)
C14—C15—H15	119.0	C4—C5—Cl2	117.72 (18)
C10—C15—H15	119.0	C6—C5—Cl2	120.17 (18)
C9—C8—C7	125.7 (2)	C1—C2—C3	118.9 (2)
C9—C8—H8	117.2	C1—C2—H2	120.5
C7—C8—H8	117.2	C3—C2—H2	120.5
C13—N1—C17	121.42 (18)	N1—C17—H17A	109.5
C13—N1—C16	120.27 (18)	N1—C17—H17B	109.5
C17—N1—C16	117.56 (18)	H17A—C17—H17B	109.5
C11—C12—C13	121.25 (19)	N1—C17—H17C	109.5
C11—C12—H12	119.4	H17A—C17—H17C	109.5
C13—C12—H12	119.4	H17B—C17—H17C	109.5
C5—C6—C1	116.9 (2)	N1—C16—H16A	109.5
C5—C6—C7	122.5 (2)	N1—C16—H16B	109.5
C1—C6—C7	120.6 (2)	H16A—C16—H16B	109.5
C4—C3—C2	121.1 (2)	N1—C16—H16C	109.5
C4—C3—Cl1	119.27 (18)	H16A—C16—H16C	109.5
C2—C3—Cl1	119.55 (19)	H16B—C16—H16C	109.5
C15—C14—C13	120.99 (19)	C2—C1—C6	122.1 (2)
C15—C14—H14	119.5	C2—C1—H1	118.9
C13—C14—H14	119.5	C6—C1—H1	118.9
			
C11—C10—C9—C8	11.4 (4)	C9—C10—C11—C12	176.6 (2)
C15—C10—C9—C8	−170.5 (2)	C9—C8—C7—O1	177.9 (2)
C11—C10—C15—C14	−0.1 (3)	C9—C8—C7—C6	−0.9 (3)
C9—C10—C15—C14	−178.3 (2)	C5—C6—C7—O1	−61.9 (3)
C10—C9—C8—C7	−176.3 (2)	C1—C6—C7—O1	117.2 (3)
C14—C13—N1—C17	−168.4 (2)	C5—C6—C7—C8	117.0 (2)
C12—C13—N1—C17	13.1 (3)	C1—C6—C7—C8	−64.0 (3)
C14—C13—N1—C16	1.6 (3)	C3—C4—C5—C6	−2.3 (3)
C12—C13—N1—C16	−177.0 (2)	C3—C4—C5—Cl2	179.29 (16)
N1—C13—C12—C11	177.7 (2)	C1—C6—C5—C4	1.5 (3)
C14—C13—C12—C11	−0.9 (3)	C7—C6—C5—C4	−179.42 (19)
C10—C15—C14—C13	1.2 (3)	C1—C6—C5—Cl2	179.91 (16)
N1—C13—C14—C15	−179.3 (2)	C7—C6—C5—Cl2	−1.0 (3)
C12—C13—C14—C15	−0.7 (3)	C4—C3—C2—C1	1.1 (3)
C2—C3—C4—C5	0.9 (3)	Cl1—C3—C2—C1	−176.93 (17)
Cl1—C3—C4—C5	178.95 (16)	C3—C2—C1—C6	−1.9 (3)
C13—C12—C11—C10	2.1 (3)	C5—C6—C1—C2	0.6 (3)
C15—C10—C11—C12	−1.5 (3)	C7—C6—C1—C2	−178.5 (2)

### Hydrogen-bond geometry (Å, °)

**Table:**

*D*—H···*A*	*D*—H	H···*A*	*D*···*A*	*D*—H···*A*
C12—H12···O1^i^	0.93	2.55	3.252 (3)	132
C4—H4···Cg1^ii^	0.93	2.95	3.784 (3)	150

Symmetry codes: (i) −*x*, *y*+1/2, −*z*+1/2; (ii) −*x*+1, *y*−1/2, −*z*+1/2.
